# COVID-19 and Teleneurology in Sub-Saharan Africa: Leveraging the Current Exigency

**DOI:** 10.3389/fpubh.2020.574505

**Published:** 2021-01-25

**Authors:** Philip Babatunde Adebayo, Olusegun John Oluwole, Funmilola Tolulope Taiwo

**Affiliations:** ^1^Neurology Section, Department of Internal Medicine, Aga Khan University, Dar es Salaam, Tanzania; ^2^Department of Neurology, King's College Hospital, Dubai, United Arab Emirates; ^3^Department of Neurology, University College Hospital, Ibadan, Nigeria

**Keywords:** COVID-19, telehealth, teleneurology, coronavirus, Africa

## Abstract

Africa has over 1.3 billion inhabitants, with over 60% of this population residing in rural areas that have poor access to medical experts. Despite having a ridiculously huge, underserved population, very few African countries currently have any form of sustained and organized telemedicine practice, and even fewer have dedicated tele-neurology services. The ongoing COVID-19 pandemic has proved to be one of the most significant disruptors of vital sectors of human endeavor in modern times. In the healthcare sector, there is an increasing advocacy to deliver non-urgent care *via* telemedicine. This paper examined the current state of tele-neurology practice and infrastructural preparedness in sub-Saharan Africa. Currently, there is over 70% mobile phone penetration in most of the countries and virtually all of them have mobile internet services of different technologies and generations. Although the needed infrastructure is increasingly available, it should be improved upon. We have proposed the access, costs, ethics, and support (ACES) model as a bespoke, holistic strategy for the successful implementation and advancement of tele-neurology in sub-Saharan Africa.

## Introduction

Coronavirus disease-2019 (COVID-19), caused by severe acute respiratory syndrome-coronavirus-2 (SARS-COV-2), broke out in the Hubei province of China in December 2019 and later became a pandemic that has now afflicted over 200 countries of the world (1). So far, COVID-19 has caused over seven million infections and over 400,000 deaths worldwide, with the highest burdens being in the United States of America (USA), selected Western European countries, and China (2). As the epidemic intensified, healthcare systems in many parts of the world rapidly became overwhelmed with severe and complicated cases of the disease for which hospitalizations and critical care are needed. Consequently, the re-deployment of healthcare workers and the repurposing of healthcare resources and facilities became a necessity in many countries to combat the existential threat posed by the virus (3, 4).

Neurology services came under severe pressure during COVID-19 due to the limited availability of neurology specialist providers and reconfiguration of existing healthcare services (4–6). Although the chronic neurology outpatient clinics were disrupted, acute services continued under the aegis of ED or neurosurgery, in those requiring critical attention (7). The situation became compounded by the widespread enforcement of lockdown measures, making it difficult for non-COVID-19 patients to access health services in places where they were still available. Furthermore, the extreme vulnerability to COVID-19 of the elderly population and those with underlying chronic medical conditions, which typify a large proportion of our neurology patients, created additional disincentives and barriers to seeking healthcare by this category of patients even when there were obvious and urgent indications for such. For example, stroke centers in Europe reported receiving much fewer acute stroke cases during this period than previously (8).

Although the full impact of the COVID-19 pandemic is yet to be evaluated in the sub-Saharan Africa (SSA) context, empirical evidence suggests a severe restriction of conventional service provision and face-to-face consultations. Few centers around the continent continue to provide neurology services via tele-neurology. Some centers with no existing tele-neurology scheme have had to develop an *ad-hoc* system that served their patient population (9). Given the low median neurologist to population ratio in Africa (0.03;100,000) compared to Europe (4.84: 100,000) (10), it is posited that tele-neurology will continue to assume increasing expansion and uptake in SSA post- COVID-19 era (11, 12). However, an estimate of the population that may benefit from tele-neurology expansion and lack of evidence for its cost-effectiveness are current gaps. Although a part of the world's second-largest continent, SSA is the region with the lowest level of economic, technological, and internet development globally, in addition to its colossal healthcare burden (13–15). In this paper, we have briefly highlighted the low-hanging fruits, and categorized them into access, costs, ethics and support (ACES) issues, which, if addressed, will improve tele-neurology practice to become a well-organized and attractive practice and service option in SSA.

## Current State of Telehealth and Tele-Neurology in Sub-Saharan Africa

The delivery of health care, health education, and health information services via remote technologies is referred to as telehealth (16). Telemedicine, often used interchangeably with telehealth, refers explicitly to the remote diagnosis and treatment of patients utilizing telecommunications technology such as telephone or exchange of information through video or images (16). Teleneurology may be defined as remote provision of neurological care, conferencing and education using various technologies to achieve connectivity, including telephone and the internet (17, 18).

Africa currently has over 1.3 billion population, with about 60% of this population residing in rural areas (19, 20). This is particularly true of sub-Saharan Africa. Despite having more people living in rural areas, medical specialists, including neurologists, are concentrated in the urban areas making access to specialist medical care difficult for most. The existing condition of healthcare service delivery in SSA is viewed as a kind of ongoing crisis-management, complete with rationing and triaging (21). This prevalent situation is occasioned by inadequate funding, inadequate personnel, inadequate training, and underserviced equipment. It is an uphill task in the light of the foregoing, to provide the desired level of quality care that SSA deserve in rural and urban settings (21).

There have been pockets of Telehealth services and Teleneurology initiatives across SSA before the pandemic. Telemedicine has been effectively utilized in other disciplines such as Opthalmology (22), HIV medicine (23), clinical psychology and psychiatry (24, 25), radiology (26), dermatology (27), neurosurgery (28), and maternal and child health (29). Although reports of organized teleneurology are limited, few teleneurology initiatives are beginning to emerge from the subcontinent. The Disease Relief through Excellent and Advanced Means (DREAM) program has incorporated a teleneurology service into its operation (30). DREAM has been operating in 11 SSA countries since 2002. The initiative provides free-to-all health services for the prevention and treatment of HIV/AIDS. The DREAM teleneurology effort proves that an established telemedicine system can launch other telehealth initiatives. In 2016, the first Arab-African teleneurology conference was held, and a “treat and teach” teleneurology initiative was recommended. The initiative aims to nurture local neurology leaders by using new telecommunication technologies to improve their knowledge and management skills while ensuring sustainability by integrating teleneurology into daily clinical practice within an existing health system using the hub and spoke model (31). One of this initiative's successes is the regular hosting of the African Movement Disorders Grand Round, a multicentre webinar series to which many African neurologists may freely connect for educational purposes (32). Most available telehealth programs deploy mobile health services to increase access to healthcare and health education (33). This model has enhanced monitoring of blood pressure control in stroke survivors (34, 35), epilepsy diagnosis, care and follow up (36), movement disorders education (37), and the care of patients with Parkinson's disease (38). Its potential role in telerehabilitation has also been evaluated (39). During the pandemic, mobile health has sustained teleconsultations (40) and remote care for epilepsy (41).

Although no randomized controlled trials have evaluated the efficacy and cost effectiveness of teleneurology in SSA, Sarfo et al. (42) had proposed the establishment of trans-continental, inter-regional, intra-regional, and national networks of neurologists to utilize teleneurology platforms to improve the reach of neurology care in SSA (42). The feasibility and acceptability of such modalities require extensive research given the different political situations, different subcontinental and national laws, healthcare as well as data sharing policies across countries in SSA. The above been said, SSA is no less prepared to utilize the advantages that teleneurology offers.

## Africa's Telecommunication Infrastructure

**Figure 1:**
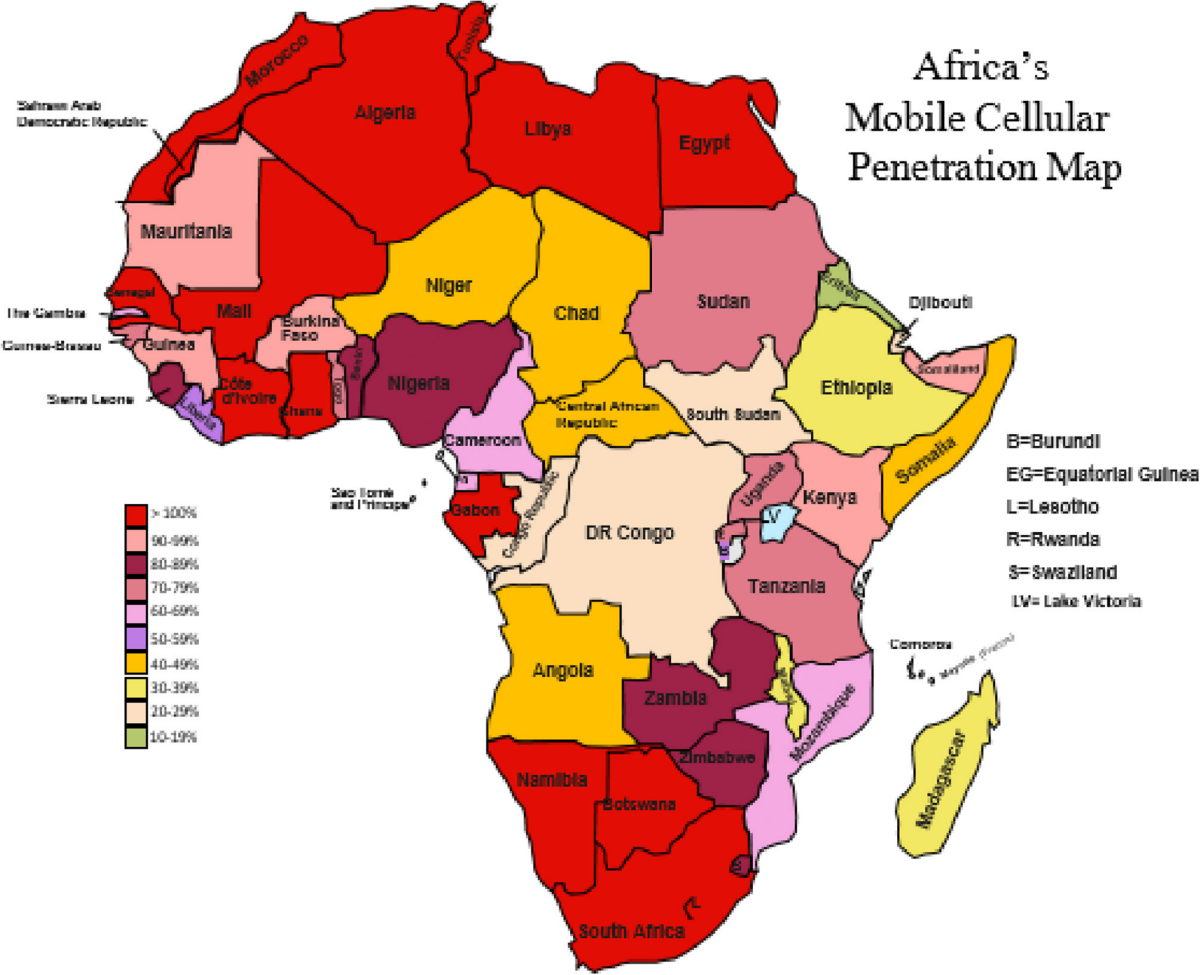
Africa's mobile cellular penetration map.

Available information reveals mobile internet as the major source of internet connectivity in most of Africa, with many countries having high mobile phone penetration, reaching up to 90% (Figure 1). By early 2019, countries such as South Africa, Botswana, Gabon, Kenya and Mauritius had well over 100% mobile phone penetration (mainly attributed to the fact that most users own more than one SIM card either from the same or different service providers) (43), though there were few countries which still had <50% penetration at that time, mostly due to political upheavals and wars (44). Such countries included Madagascar, Malawi, and Sudan (16). Mobile internet services are dispensed or sold to citizens in monthly/weekly data plans that are separate from calling plans. The mobile internet connections are often not available in rural communities. Also, not all of the cities where the connections exist have access to broadband internet. In many of such cities in SSA, the governments and internet service providing companies are still in the process of either upgrading outmoded existing infrastructures to provide broadband capabilities or expanding them to provide some form of basic low bandwidth internet connectivity in places where they have not yet existed.

Nigeria currently has broadband coverage of about 30% and plans to increase it to 70% by 2021. Ghana also recently began to implement an upgrade from 2G low bandwidth to 3G (44). In the cities where mobile broadband has become available, high pricing and congestion remain as obstacles. The situation appears different in northern Africa, with Egypt having a decent coverage of fixed-line broadband internet connectivity in homes and offices in addition to ubiquitous mobile internet available for personal use (44). Similarly, Tunisia is reputed as having one of the most sophisticated telecommunications and broadband infrastructures in North Africa. Outside of northern Africa, there is evidence that South Africa is making increasing strides in the area of fixed-line broadband (44).

Although broadband internet is still not universally available in all countries in Africa, the fair availability of modest internet technology coupled with decent mobile penetration provides the opportunity to improve telehealth and indeed, tele-neurology practice. Certainly, some types of teleneurology practices such as remote patient monitoring (RPM) might be impracticable in rural communities where the internet connectivity and the available technologies are too basic or weak to support the required mobile applications.

The model of transcontinental and transnational tele-neurology approach proposed by Sarfo et al. (42), has already been practiced by other disciplines. For example, Wamala et al. reveals the existence of some form of organized telepathology services in Uganda (33), as well as organized telemedicine and tele-education programs in Bamako, Mali which had internal collaborations with Geneva, Switzerland (45). Furthermore, there is evidence of a growing structured and regulated telemedicine practice in South Africa (46). Interestingly also, there is a recently implemented pilot telemedicine initiative in maternal and child health in Nigeria covering few handpicked states in the country (47).

## Implication of COVID-19 Pandemic for Telehealth and Teleneurology in Sub-Saharan Africa

Before this era in SSA, the bourgeoning use of telehealth has been found to have both clinical and educational significance in a way similar to high-income countries (HIC) within the disciplines that have deployed telemedicine services and in movement disorders (32, 37, 48). The COVID-19 pandemic has created undeniable and unprecedented difficulties with continuing to offer non-urgent, but certainly needed, care to a large group of our patients, including those with neurological disorders. Even after the lockdown measures are relaxed as they are beginning to, the sparse distribution of newly lauched vaccines against Sars-Cov-2 might continue to deter in-hospital care. To bridge this gap, many medical societies and associations worldwide are encouraging the adoption of telemedicine and telehealth to meet the needs of patients whose care does not necessitate direct physician contact. For example, the Association of British Neurologists (ABN) has recently published guidelines on how to establish teleneurology services during the COVID-19 pandemic effectively. In this guideline, the ABN encourages all neurology consultations to be done via telemedicine except in cases where in-person consultation is unavoidable (49).

The pandemic has undoubtedly provided an opportunity to adopt tele-neurology as a viable addition or alternative to conventional neurological practice. An obvious drawback to teleneurology is the inability to touch the patient or perform some neuro-examination aspects. The examination will need, therefore, to rely on visual inspections with the patient's assistance (49). Apart from the potential to reduce cost and improve outcomes, the real value of tele-neurology in this era lies in its ability to prevent the spread of and the exposure to potential Sars Cov-2 infection. Anecdotal report from Kenya suggests a good uptake and responsiveness to a new tele-neurology service initiated in response to the COVID-19 pandemic (9). In the same vein, the Health Professions Council of South Africa (HPCSA) had to amend the pre-existing requirements for telehealth in response to the pandemic. Now, physicians in South Africa do not need to have had prior in-person consultation with a patient before such a patient could be seen *via* telemedicine (46).

Going forward, institutions in SSA would need to progressively integrate an organized teleneurology practice into their neurology service delivery. We are proposing the ACES (Access, Cost, Ethics, and Support) framework, which attempts to put into clear perspective, the adaptable core scalable goals which institutions and telehealth stakeholders in SSA can work with. We acknowledge that political will, training, education, advocacy, and the continued level of interest in telehealth beyond COVID-19 by the different national health regulatory authorities, will play an essential role in determining whether telehealth and indeed tele-neurology will assume a different pedestal in the subcontinent.

## ACES Strategic Focus

**Figure 2:**
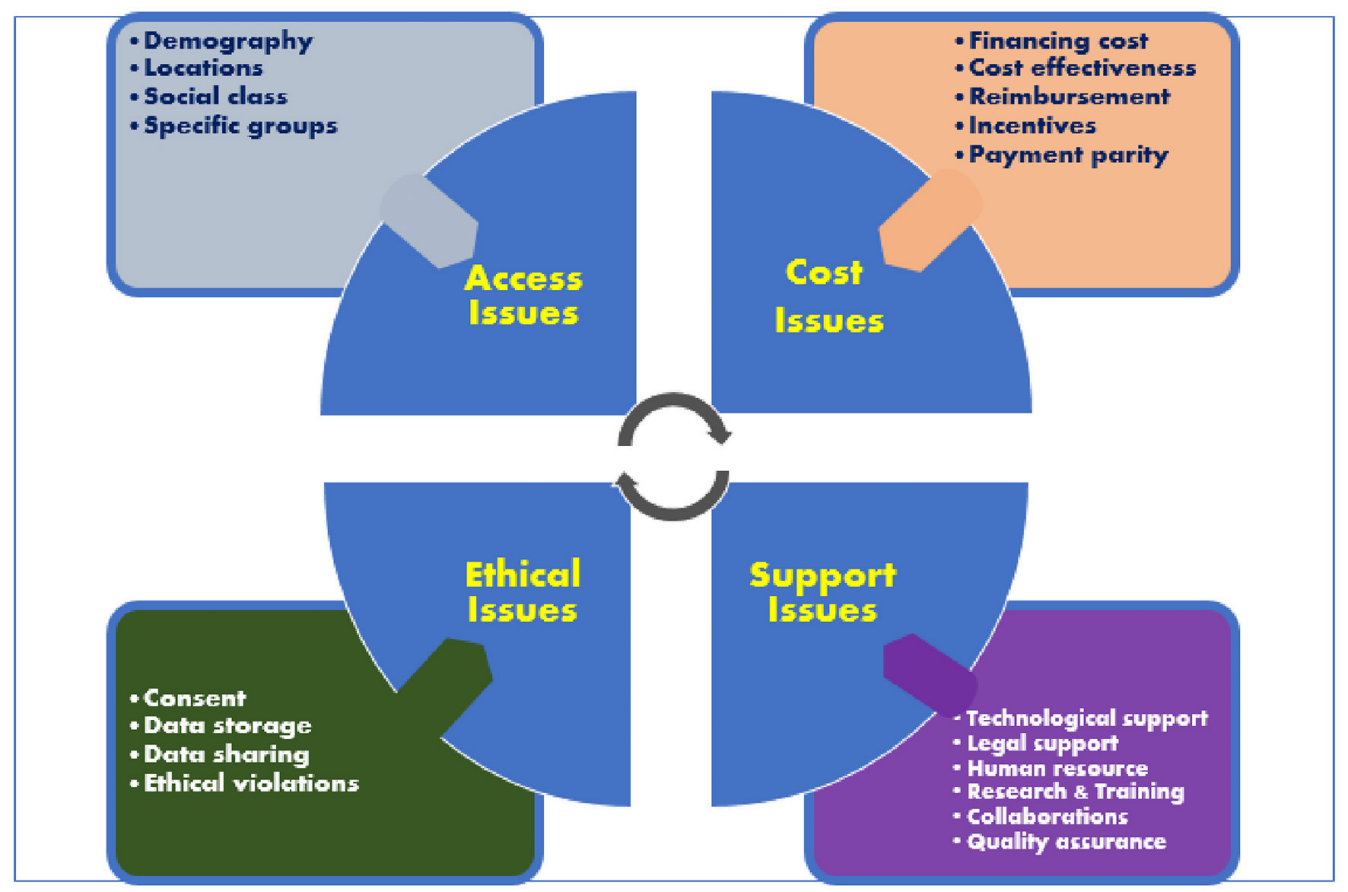
ACES model for expansion of teleneurology services in Sub-Sahran Africa.

The COVID-19 pandemic has raised pertinent questions about the delivery of health care even in urban societies. It is posited that telehealth and tele-neurology would increase with time. Currently, in SSA, tele-neurology faces a plethora of challenges such as dependency on funding, unclear healthcare system responsibilities and shortage of neurologists as well as non-physician health workers (NPHW) to provide care, leadership, and drive. In addition to the aforementioned factors, inadequate and unreliable infrastructural support for telehealth services significantly limits tele-neurology in SSA (42). Although we did not estimate the population that can potentially benefit from tele-neurology services in SSA, emerging evidence reveals that the potential for tele-neurology uptake is enormous. For example, Mcginley et al. reported a 533% increase in the usage of a tele-neurology service for all patients in an academic institution during COVID-19 era (50). The ACES model (Figure 2) summarizes our idea of driving new tele-neurology services beyond the COVID-19 period.

### Access

To be effective and far-reaching, tele-neurology services in SSA will have to consider the demography and location of the population that it is meant to serve. Efforts to include all social classes and specific groups of people (for example, vulnerable groups) will facilitate service uptake and promote equity in access. Tele-neurology service model will have to be inter-regional and intra-regional as proposed by Sarfo et al. (42) but cannot be limited to academic institutions because there are neurologists and neurological service delivery outside of academic centers. According to the world federation of neurology (WFN) survey, only a small proportion (0–25%) of neurologists work full time in academic centers. A substantial majority (75–100%) of neurologists also work in private practice in many countries, including those in Africa (51). Using the stroke medicine model, African healthcare institutions may employ the *hub-and-spoke system*—a structure of telemedicine in which a certified comprehensive stroke center, usually in a large urban area, serves as the primary stroke center (the hub). The spokes are in remote areas, generally smaller regional rural or underserviced hospitals. In the context of tele-neurology, the neurology specialists at the hub (an institution of health) will consult with doctors and people with neurologic symptoms at the remote sites (spokes) (52). The principles of task-shifting may be blended in this endeavor because health system development is still not even across SSA (53). The novel strategy of task-shifting has been shown to build relevant competencies among NPHW to bridge the human resource gap in the provision of services for non-communicable diseases such as HIV/AIDS and stroke. Our earlier reported experience with task-shifting for stroke recognition and care in southwestern Nigeria (53) indicates that this model is replicable for tele-neurology. The NPHW will require training and education to recognize common neurological disorders at remote sites and to co-ordinate with neurology specialists at the hub.

### Costs

Although some extant literature has suggested that tele-neurology is not more cost-effective compared to conventional neurology service, especially with teleconsultation (54, 55), its long-time cost-effectiveness has now been established (56, 57). In most SSA countries, where a significant proportion of the population are not covered by health insurance, new tele-neurology services will need to demonstrate that people can get quality telehealth and tele-neurology services at cost-effective rates. Tele-health vendors will need to consider financing costs while institutions will need to ensure adequate remuneration and pay parity for physicians. While some schools of thought argue that telehealth and, by extension, tele-neurology services should be free-of-charge to be ethical and equitable, this healthcare delivery model is not without its challenges. Instead, a cost-effective model is advocated by other schools of thought (52). A free-of-charge tele-neurology service, if not adequately funded, can impede innovation in healthcare provision because such designs may not be incentivized. Besides, insufficient medical personnel staffing and a skewed doctor-patient ratio (a result of inadequate budgetary allocation and brain drain) can reduce care quality. For this model to be sustainable, countries in SSA need to increase the budget for health and retain sizeable professional stakeholders involved in tele-neurology service provision. In the context of improved budgetary allocation to health, the benefits of free-of-charge tele-neurology services can be realized. The gains of free-of-charge telehealth services include increase accessibility and affordability, an expanded range of tele-neurology services, and improved health-seeking behavior of the populace (58).

### Ethics Issues

Privacy and data confidentiality will begin to take a central place as tele-neurology services advance. The current stakeholders and new service providers will need to address data storage (store and transfer) issues. Current regional or national data protection policies should be standardized and made applicable to tele-neurology services. Matters that may constitute a conflict-of-interest conundrum between stakeholders would need clear definitions from the outset. Data storage, sharing, and forwarding would need to be governed by serious ethical guidelines. New tele-neurology programs must court patient's confidence by having a robust privacy and security plan that should be communicated to the patients (59).

### Supports

All the stages of tele-neurology development require adequate support. The preparatory stage requires adequate support for and by the stakeholders which is made up of a multidisciplinary team (administrative staff, neurologists, other specialists, policymakers, telemedicine vendors, and ICT maintenance team). Telemedicine vendors would need to ensure network coverage in geographically isolated rural areas. Policymakers would need to be convinced that investments in telemedicine technology, improved connectivity, infrastructure, and network personnel are justified. Legal support is equally required, as are other supports provided by data-driven initiatives that research, development, and training stimulate. Finally, standards would need to be created for the engagement, training, and supervision of tele-neurology providers like what obtains in conventional practice to assure quality assurance and system organization. This multifaceted support system will ensure the sustainability of new tele-neurology services in SSA (60).

## Conclusion

Certainly, telemedicine and tele-neurology services are still in their infancy in SSA. The exigency created by the COVID-19 pandemic is a wake-up call to institutions, countries, and indeed, heath authorities in SSA to redefine their posturing to telehealth, including tele-neurology. The proposed ACES model may ensure the sustainability of tele-neurology services in the SSA region beyond COVID-19. Improvement in systemic issues such as infrastructure and political will would play a key role in building and sustaining tele-neurology services in the SSA subcontinent (5, 30).

## Data Availability Statement

The original contributions presented in the study are included in the article/supplementary material, further inquiries can be directed to the corresponding author/s.

## Author Contributions

PA conceived the idea of the manuscript. PA, OO, and FT drafted the manuscript. All authors reviewed the manuscript for intellectual content and approved the final manuscript.

## Conflict of Interest

The authors declare that the research was conducted in the absence of any commercial or financial relationships that could be construed as a potential conflict of interest.
